# Feasibility of MR Metabolomics for Immediate Analysis of Resection Margins during Breast Cancer Surgery

**DOI:** 10.1371/journal.pone.0061578

**Published:** 2013-04-17

**Authors:** Tone F. Bathen, Brigitte Geurts, Beathe Sitter, Hans E. Fjøsne, Steinar Lundgren, Lutgarde M. Buydens, Ingrid S. Gribbestad, Geert Postma, Guro F. Giskeødegård

**Affiliations:** 1 Department of Circulation and Medical Imaging, Faculty of Medicine, The Norwegian University of Science and Technology (NTNU), Trondheim, Norway; 2 St. Olavs University Hospital, Trondheim, Norway; 3 Radboud University Nijmegen, Institute for Molecules and Materials, Nijmegen, The Netherlands; 4 Department of Technology, Sør-Trøndelag University College, Trondheim, Norway; 5 Department of Surgery, St. Olavs University Hospital, Trondheim, Norway; 6 Department of Oncology, St. Olavs University Hospital, Trondheim, Norway; 7 Department of Cancer Research and Molecular Medicine, Faculty of Medicine, The Norwegian University of Science and Technology (NTNU), Trondheim, Norway; Texas A&M University, United States of America

## Abstract

In this study, the feasibility of high resolution magic angle spinning (HR MAS) magnetic resonance spectroscopy (MRS) of small tissue biopsies to distinguish between tumor and non-involved adjacent tissue was investigated. With the current methods, delineation of the tumor borders during breast cancer surgery is a challenging task for the surgeon, and a significant number of re-surgeries occur. We analyzed 328 tissue samples from 228 breast cancer patients using HR MAS MRS. Partial least squares discriminant analysis (PLS-DA) was applied to discriminate between tumor and non-involved adjacent tissue. Using proper double cross validation, high sensitivity and specificity of 91% and 93%, respectively was achieved. Analysis of the loading profiles from both principal component analysis (PCA) and PLS-DA showed the choline-containing metabolites as main biomarkers for tumor content, with phosphocholine being especially high in tumor tissue. Other indicative metabolites include glycine, taurine and glucose. We conclude that metabolic profiling by HR MAS MRS may be a potential method for on-line analysis of resection margins during breast cancer surgery to reduce the number of re-surgeries and risk of local recurrence.

## Introduction

Cancer is a major cause of death, with incidences predicted to increase with the aging population [1]. Breast cancer is the most common malignancy in women, and annually nearly 3000 women receive surgery and additional treatment for breast cancer in Norway [2]. In order to minimize the risk of local recurrences, infiltrating tumors should be removed with free resection margins. Currently, resection margins are evaluated by a pathologist after surgery, and a significant number of patients is scheduled for re-surgery [3], [4]. In addition to the increased burden for the patient, this also has a cost and capacity downside for the hospital. Providing information to distinguish between tumor and non-involved adjacent tissue during breast cancer surgery can help surgeons delineate the tumor margins more accurately, thereby significantly reducing the number of necessary re-surgeries.

The term metabolomics refers to the systematic studies of small-molecular compounds of metabolism in cells, biofluids, organs or tissues [5]. Metabolites are downstream products of metabolism, and thereby a close measure of the phenotype of the biological system being studied. Magnetic resonance spectroscopy (MRS) has a long tradition for metabolite analyses, and the use of high resolution magic angle spinning (HR MAS) enables analyses of intact tissue samples [6]. This methodology is a promising tool within cancer diagnostics and treatment evaluation [7], and has already been applied in many studies of cancer [8]–[17]. Numerous metabolites are detected in breast cancer biopsies, and the spectral quality achieved using HR MAS is comparable to what is obtained with liquid extracts [18]. The metabolic information contained in the spectra can be used to establish prognostic and predictive classifiers using appropriate multivariate statistical analyses, such as principal component analysis (PCA) and partial least squares (PLS) regression, which handles the highly co-variant nature of MRS variables.

Recent technological advances facilitate automated analyses of biological samples, and installations of MR equipment in close proximity to the surgical theaters are in a growing phase. A case report from colon adenocarcinoma supports that the time-response of HR MAS is sufficiently fast for effective use on-line during surgery [19]. Metabolic profiling thus has the potential to become a method for rapid characterization of cancerous biopsies in the operation theatre. Previous studies have shown the ability of HR MAS to distinguish between cancerous and normal cervical [20], colon [15], [19] and prostate tissues [16], [21]. A study using ultrasound-guided breast core-needle biopsies was recently published [22]. However, the reported sensitivity for predicting cancer was low, possibly due to the low number of included samples. Furthermore, potential over-fitting due to multiple samples from single subjects was not considered.

The aim of the current study was to evaluate the accuracy of HR MAS MRS derived classifiers to distinguish breast tumor and non-involved adjacent tissue for future on-line analyses within the surgical theater using biopsies from a large biobank. For this purpose, a robust validation scheme handling multiple samples from single subjects has been implemented. Furthermore, the classification impact of biopsies with low tumor content has been investigated.

## Methods

### Patients and Tissue Samples

Cancer and non-involved tissue from breast cancer patients undergoing surgery at St.Olavs Hospital, Trondheim, Norway and Molde Hospital, Molde, Norway, have been consecutively enrolled in a local biobank. The tissue samples are immediately frozen in liquid nitrogen and stored until the MR analysis. The current study includes 328 tissue samples from 228 patients with surgery performed between 1999 and 2006. None of the patients received neoadjuvant treatment prior to surgery. Information on diagnosis, tumor grade, hormone receptor status, and lymph node involvement was obtained from patient records, including pathology reports. Axillary lymph node status was examined by sentinel node procedure or axillary clearance. Histological grade was determined according to guidelines of the Norwegian Breast Cancer Group (NBCG), based on the Bloom and Richardson classification system [23]. Estrogen receptor (ER) and progesterone receptor (PgR) status were determined by immunohistochemistry (≥10% staining cancer cells considered receptor positive). Detailed patient characteristics are described in Table 1. The study was approved by the Regional Committee for Medical and Health Research Ethics, Central Norway, and written informed consent was obtained from all included patients.

**Table 1:** Patient characteristics.

**Age**	Median (range)	60.7 (29.6–91.8)
**Tumor size**	Median (range)	2.0 (0–7)
**Diagnosis**	IDC	175
	ILC	21
	Other*	32
**Grade**	1	26
	2	100
	3	80
	Unknown	22
**ER** ** **status**	Positive	168
	Negative	49
	Unknown	11
**PgR** *** **status**	Positive	132
	Negative	80
	Unknown	16
**Lymph node status**	Positive	90
	Negative	122
	Unknown	16

* Ductal carcinoma in situ, mucinous carcinoma, several diagnoses.

** Estrogen receptor status.

*** Progesterone receptor status.

### HR MAS MRS

HR MAS MRS analyses of the tissue samples (n = 328) were performed on a Bruker Avance DRX600 spectrometer (Bruker BioSpin GmbH, Germany) equipped with a ^1^H/^13^C MAS probe with gradient aligned with the magic angle axis. Frozen tissue samples were cut to fit a MAS rotor (50 μL, median sample weight 16.2 mg) added phosphate buffered saline (PBS, 40 μL) based on D_2_O containing trimethylsilyl 3-propionic acid sodium salt (TSP, 1.0 mM). Samples were spun at 5 kHz at a temperature of 4°C. Proton spectra were acquired using a spin-echo Carr-Purcell-Meiboom-Gill (CPMG) sequence (cpmgpr, Bruker) with 2 s water suppression prior to a 90° excitation pulse. T_2_ filtering was obtained using a delay of 1 ms repeated 136 times, resulting in an effective echo time of 285 ms. A total of 128 scans over a spectral region of 10 kHz were collected into 32 k points, giving an acquisition time of 1.64 s. The spectra were Fourier transformed into 128 K after 0.3 Hz exponential line broadening, and chemical shifts were calibrated according to TSP (0 ppm). The tissue specimens were fixed in 10% formalin and embedded in paraffin after the HR MAS analysis. One 5 µm section was cut from each paraffin block, stained with haematoxylin, erythrosine, and saffron (HES), and examined microscopically by an experienced pathologist. The relative areas of normal and neoplastic epithelial elements were scored visually. An overview of the biopsies and estimated tumor content is given in Table 2.

**Table 2:** Tissue composition of the biopsies for the study cohort.

	Number of samples/patients	Median (min-max) distribution of tissue type (%)				
		Tumor	Fat	Connective	Glandular	Necrotic
Normal adjacent tissue (Tumor content 0% )	65/43	0	20(0–100)	60(0–100)	0(0–40)	0
Tumor content 1–4%	5/5	2(2–4)	5(0–98)	78(0–93)	0(0–15)	0(0–32)
Tumor content 5%	32/29	5	0 (0–47.5)	95 (47.5–95)	0(0–20)	0 (0–5)
Tumor content >5%	226/182	20 (7–95)	0(0–80)	70(0–93)	0(0–20)	0(0–30)

### Data Preprocessing

The spectral region between −0.08 and 4.7 ppm was selected for further processing. Values of negative spikes were replaced by boundary values and the baseline offset was corrected by subtracting the lowest value. Baseline trends were removed by asymmetric least squares [24] with the smoothing parameter λ = 1e7, the asymmetry parameter p = 0.0001 and the order of differences in penalty d = 2. Peak alignment was performed using icoshift [25] with 39 manually chosen intervals and the highest correlated spectrum as the reference as described in ref. [26]. The area upfield from 3.0 ppm was removed after preprocessing. Signals from ethanol pollutions at 3.691–3.642 ppm were deleted together with fatty acid residuals at 4.200–4.400 ppm, resulting in spectra of 2759 variables. The resulting spectra were mean-normalized. Preprocessing of the data was performed in Matlab 7.6.0.

### Multivariate Data Analysis

The variation of the data was explored by PCA [27]. PLS discriminant analysis (PLS-DA) [28] was used to discriminate cancer samples from adjacent non-involved tissue. PLS-DA was executed after variable stability (VAST) scaling [29] of the data in a supervised manner, with mean-centering instead of autoscaling prior to multiplication of the scaling weights. The classification performance was obtained using double cross-validation [30] consisting of two nested leave-20%-out cross-validation loops. The inner loop (repeated 20 times) was used to optimize the number of latent variables (LVs) for PLS-DA, while the outer cross-validation loop (repeated 80 times) was used to determine the classification performance (accuracy, sensitivity and specificity). In order to circumvent overoptimistic results it was assured that data from the same patient were always present in one set, either the training, test, or validation set. VAST scaling was applied during each cross-validation loop on the training set and the resulting scaling parameters were applied independently to the test or validation set. The loadings of the PLS-DA models were colored according to their variable importance in projection (VIP) scores [31]. Further validation of the significance of the PLS-DA classification results were performed by permutation testing, and p-values <0.05 were considered significant [32].

Alternative ways of PLS-DA model making were investigated by handling samples with very low tumor content in different ways; either by defining samples with a low tumor cell content (between 0–4%) as adjacent non-involved tissue, or by defining all samples with tumor cell content >0% as tumor tissue. Models were also made by removing samples with low tumor cell content from the training data and including them only in the test set (Table 3).

**Table 3:** PLS-DA classification results for separating tumor and adjacent non-involved tissue.

	Correct classification	Sensitivity	Specificity
Tumor (≥5%TC) vs non-involved (<5% TC)	90%	83%	92%
Tumor (>0% TC) vs non-involved (0% TC)	89%	87%	89%
Tumor (>0% TC ) vs non-involved (0% TC)*	92%	91%	93%
Tumor (>0% TC) vs non-involved (0% TC)**	92%	90%	94%

* Samples with 0%<tumor content <5% not used for model training, but included in testing only (5 samples, see Table 2).

** Samples with 0%<tumor content ≤5% not used for model training, but included in testing only (5+32 = 37 samples, see Table 2).

TC, tumor content.

As an additional approach, classification was performed using only the spectral region containing the choline-containing metabolites (3.252–3.196 ppm) as input for the classification model. This approach is relevant for the ongoing discussion concerning choline metabolism in cancer.

PLS-DA was performed using the PLS_Toolbox 6.5.1 for MATLAB (Eigenvector Research, Inc. Wenatchee, WA).

## Results

**Figure 1:**
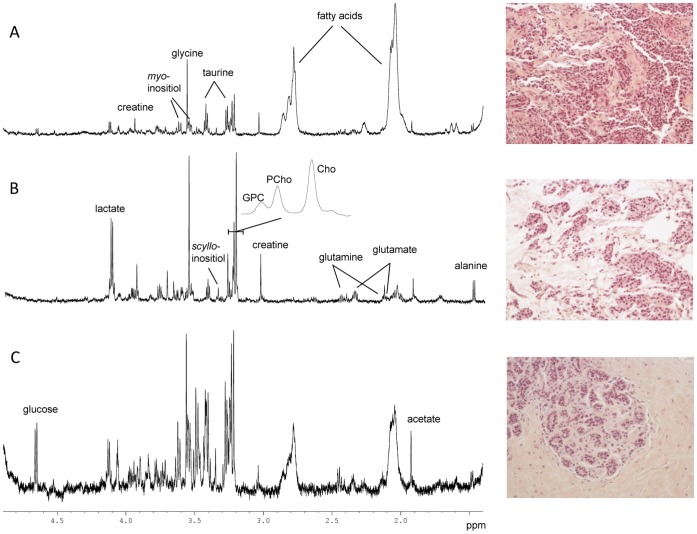
HR MAS spectra and illustration of typical features observed in the corresponding HES images (200X). (A) Invasive ductal carcinoma with an estimated tumor content of 80% in the analysed biopsy. (B) Invasive mucinous carcinoma with an estimated tumor content of 60% in the analysed biopsy. (C) Normal breast tissue (adjacent to tumor). No tumor cells were detected, and the HES image shows the typical feature of normal terminal lobular duct units. The poor signal to noise ratio in this spectrum is probably due to the high level of connective tissue (85%).

Representative HR MAS MR spectra from breast biopsies with high tumor cell content and normal adjacent tissue are given in Figure 1. As previously reported [14], adipose tissue has an immense impact on the spectral features due to the methylene and methyl lipid protons giving rise to large signals centred around 1.3 and 0.9 ppm, respectively. None the less, the low molecular weight metabolites are still visible in the spectra due to the T2-filtering applied for MR acquisition.

**Figure 2:**
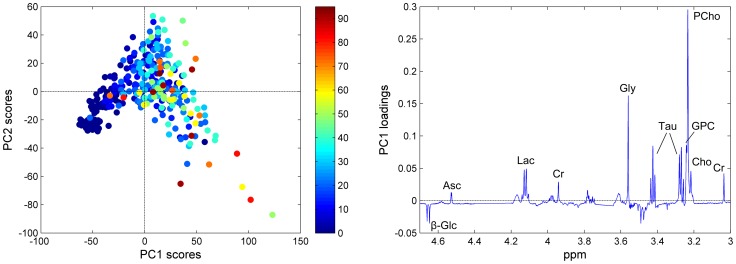
Variation in tumor cell content as described by PCA. (A) The score plot of the pre-processed spectra, colored according to the tumor cell content (%) of the corresponding biopsies. (B) The corresponding loading profile of PC1, explaining 40.1% of the total variation of the data. β-Glc, β-glucose; Asc, ascorbate; Lac, lactate; Cr, creatine; Gly, glycine; Tau, taurine; GPC, glycerophosphocholine; PCho, phosphocholine; Cho, free choline.

A PCA score plot of the preprocessed spectra coloured according to the tumor cell content of the samples is presented in Figure 2A. A trend related to increasing tumor content is visible from left to right along the first principal component (PC1, explaining 40.1% of the variance of the spectra), showing that differences in tumor content are contributing to the main variation of the data set. The corresponding loading profile (Figure 2B) shows that the samples with a low tumor content have higher levels of glucose, while samples with a high tumor content have higher levels of ascorbate, lactate, creatine, glycine, taurine and the choline-containing metabolites (glycerophosphocholine (GPC), phosphocholine (PCho), and free choline). The second PC, explaining 16.3% of the variation, is separating samples based on their level of PCho with a low PC2 score representing high levels of PCho (not shown). Upon visual inspection, PC3 and higher do not seem to explain any variance related to tumor content.

**Figure 3:**
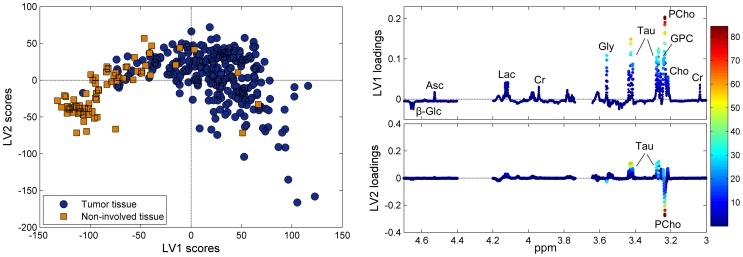
PLS- DA classification of tumor and non-involved tissue. (A) The score plot, separating tumor and non-involved tissue, and (B) the corresponding loading profiles of LV1 and LV2. LV1 and LV2 explain 35.8% and 13.2% of the x-variation and 53.3% and 4.9% of the y-variation, respectively. β-Glc, β-glucose; Asc, ascorbate; Lac, lactate; Cr, creatine; Gly, glycine; Tau, taurine; GPC, glycerophosphocholine; PCho, phosphocholine; Cho, free choline.

The score plot from PLS-DA (Figure 3A) shows clear clusters of normal adjacent tissue and cancerous tissue. The distinction is mainly due to the variation described by LV1, attributed to higher levels of ascorbate, lactate, creatine, glycine, taurine, and the choline-containing metabolites in addition to lower levels of glucose in the cancerous samples, a pattern similar to the one observed by PCA. The distinction is less clear on LV2, which is mainly attributed to variation in taurine and PCho levels among the whole sample cohort. The PLS-DA classification results for separating tumor and non-involved tissue are given in Table 3. Various schemes for classifying the tumor and non-involved adjacent tissue were investigated. Nearly all approaches lead to accuracy, sensitivity and specificity around 90%. The best classification result is achieved when defining all samples containing tumor cells (>0% tumor content) as tumor tissue, and training the classification model leaving out biopsies with a low tumor content (0%<tumor content <5%). All classification results were highly significant (p<0.001 by permutation testing).

**Figure 4:**
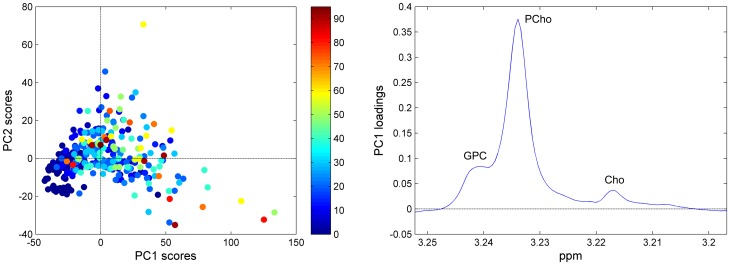
Variation in tumor cell content as described by PCA of the choline region. (A) The score plot of the choline-containing metabolite region of the spectra, colored according to the tumor cell content (%) of the corresponding biopsies. (B) The corresponding loading profile of PC1 explaining 71.3% of the total variation of the data.

Choline metabolism has a central role in breast cancer research. Figure 4 shows the PCA score and corresponding loading (PC1) plot from analysis of the choline spectral region (3.252–3.196 ppm), including only the signals from GPC, PCho, and choline. The samples are labelled according to the tumor cell content (%). In general, a high score for PC1 is associated with high tumor content, and as shown by the loading profile, this is attributed to relatively higher values of all the choline-containing metabolites, especially PCho. 71.3% of the variation of the spectra is explained in the first PC. PLS-DA modelling shows that the choline region contains enough information to significantly discriminate tumor and non-involved adjacent tissue (p<0.001 by permutation testing). However, the classification result reporting accuracy, sensitivity and specificity of 88%, 87% and 88%, respectively, are lower than when including the complete low molecular weight metabolic profile.

## Discussion

In this study, we have proven the possibility to differentiate breast tumor and non-involved tissue with high sensitivity and specificity based on metabolic profiling by HR MAS MRS. Using different approaches for classification, we showed accurate discrimination of tumor and non-involved tissue also for samples with low tumor cell content. This shows the possibility of applying MR metabolomics for real-time determination of resection margins during breast cancer surgery.

MR metabolomics has the advantage of being a rapid and low-cost analysis method that can be performed while the patient is still on the surgery table. The total process of preparing and analysing the biopsy takes approximately 15 minutes, while data analysis of the resulting spectra using previously developed and validated classification models can be performed in under a minute. A free resection margin determined during surgery can then be further validated by histology after surgery. Due to the non-destructive nature of HR MAS MRS, this can even be done on the same tissue sample after HR MAS.

Tumor samples were shown to contain higher levels of ascorbate, lactate, creatine, glycine, taurine, and the choline-containing metabolites, in addition to lower levels of glucose compared to non-involved tissue, and this metabolic pattern was associated with increasing amounts of tumor cells present in the sample. An increased consumption of glucose and high accumulated levels of lactate in tumor samples are consistent with the Warburg effect [33], [34]. Further, we showed that tumor and non-involved tissue could be accurately discriminated using only the choline-region as input for the classifier. The choline-containing metabolites are of high interest in cancer research, and abnormal choline metabolism is frequently observed [35]. Samples with high tumor cell content had higher levels of all the choline-containing metabolites compared to samples with low tumor cell content, with PCho being especially elevated in tumors. This is reflecting the increased proliferation rate in tumors, as choline is an important constituent of cell membranes through the formation of phosphatidylcholine, and PCho and GPC are precursors and breakdown products of this activity [36]. In some cancer cells, a relatively large amount of glycolytic carbon is diverted into serine and glycine metabolism through phosphoglycerate dehydro-genase (PHGDH), which could explain the increased glycine concentration in the samples containing tumor cells [37]. The metabolic reprogramming in cancer is comprehensive, and it is reasonable that other metabolites such as creatine and taurine also are affected. This may explain the observed multi-collinearity of the metabolite changes.

This study was performed using a large patient cohort, with a robust validation scheme correcting for the use of several samples from the same patient. This step is important as several biopsies from each patient will be analysed for determination of surgical margins in a clinical setting. A motivation for investigating the effect of various options of treating tissue samples with a low tumor content was that these samples frequently in literature are removed from the data set [38], [39]. This is, however, not an optimal approach if one wants to detect non-involved adjacent tissue; even with the presence of very few tumor cells, a sample will be considered tumor tissue in a clinical setting. The optimal classification procedure was achieved when removing the samples with low tumor cell content from model training, and including these samples as tumor samples in the test data. A sensitivity and specificity of 91% and 93%, respectively, were achieved. However, only five spectra had tumor cell content between 1–4%, while 37 spectra had tumor cell content between 1–5%. Inclusion of more samples with low tumor cell content is desirable in order to truly test the prediction performance on such samples, as it might be expected that tumor tissue close to the margins may contain a low number of tumor cells.

Breast tissue biopsies contain a varying amount of lipids, and the lipid signals present at 1.3 and 0.9 ppm were in many cases the most intense signals in the spectra despite using a lipid-suppressing CPMG sequence for MRS acquisition. In order to circumvent dominance of the lipid signals during data analysis, the regions containing these signals were removed from the spectra and the data were normalized to equal total intensity prior to analysis. Thus, the differing amount of breast lipids present in the samples is partly corrected for, and emphasis is made to the low molecular weight metabolites.

We have previously shown that the MR metabolic profile of a tissue sample contains prognostic information beyond that of traditional clinical parameters, with high levels of lactate and glycine being indicative of lower 5-year survival rates [39], [40]. Furthermore, the MR metabolic profile contains information related to breast cancer subtypes [41], [42]. In addition, MR metabolic profiles have been correlated to hormone receptor status and lymphatic spread [8], [22], [38]. This renders the possibility to use the spectra acquired during surgery for prognostic assessment of patients for further treatment planning after surgical removal of the tumor.

For further assessment of using MR metabolomics as a clinical tool for determining surgical margins, differentiation of cancer and non-involved tissue should be examined by real-time analysis of surgical biopsies from tumor border region, and the results should be compared to histology. A more quantitative histopathology assessment of the tumor content should be performed, by investigating multiple sections throughout the biopsy after HR MAS analysis. The effect of using either frozen or completely fresh tissue should be investigated, as a study comparing the metabolic profiles of fresh tissue to over-night freezing have shown moderate changes in the metabolite concentrations due to freezing [43]. It is possible that even better separation of cancer and non-involved tissue could be achieved by using completely fresh, non-frozen tissue samples.

### Conclusion

Based on a large patient cohort (228 patients) we have shown that metabolic profiling by HR MAS MRS can be used for accurate classification of tumor and non-involved adjacent breast tissue. The analysis has a time frame enabling on-line analysis of resection margins during breast cancer surgery. The results were obtained using a proper double cross validation procedure assuring that the data originating from the same patient always were present in one set, either the training, test, or validation set. Moreover, using different visualization techniques we were able to identify the metabolites related to the differentiation of tumor and non-involved adjacent tissue.
